# Validity of claims made in weight management research: a narrative review of dietetic articles

**DOI:** 10.1186/1475-2891-9-30

**Published:** 2010-07-20

**Authors:** Lucy Aphramor

**Affiliations:** 1 Applied Research Centre in Health & Lifestyle Interventions, Faculty of Health & Life Sciences, Coventry University, Priory St., Coventry, CV1 5FB, UK

## Abstract

**Background:**

The best available evidence demonstrates that conventional weight management has a high long-term failure rate. The ethical implications of continued reliance on an energy deficit approach to weight management are under-explored.

**Methods:**

A narrative literature review of journal articles in *The Journal of Human Nutrition and Dietetics *from 2004 to 2008.

**Results:**

Although the energy deficit approach to weight management has a high long-term failure rate it continues to dominate research in the field. In the current research agenda, controversies and complexities in the evidence base are inadequately discussed, and claims about the likely success of weight management misrepresent available evidence.

**Conclusions:**

Dietetic literature on weight management fails to meet the standards of evidence based medicine. Research in the field is characterised by speculative claims that fail to accurately represent the available data. There is a corresponding lack of debate on the ethical implications of continuing to promote ineffective treatment regimes and little research into alternative non-weight centred approaches. An alternative health at every size approach is recommended.

## Background

The assessment and management of body weight is a major preoccupation of contemporary UK health policy. Clinical interventions focus on achieving energy balance deficit and are premised on claims that excess weight/fatness (body mass index (BMI) > 25) is a significant direct cause of morbidity and mortality and, correspondingly, that weight loss in fat ('overweight' or 'obese') people will reduce risk and/or improve health outcomes. Yet at the same time, reputable organisations continue to urge caution with regard to the evidence base presumed to underpin these tenets. Thus, epidemiological data from the Center for Disease Control highlights that "even severe obesity failed to show up as a statistically significant mortality risk" and confirmed previous findings that 'overweight' people live slightly longer than people of 'healthy' weight [1]. In addition, there is a growing inter-disciplinary literature [2] that contests the effectiveness of a weight-centred approach to health and draws attention to the ethical implications of unintentional outcomes. The vigorous scientific debate on approaches to healthy weight is inadequately represented in research. It could be said that weight loss enjoys special immunity from accepted standards in clinical practice and publishing ethics. This paper analyses a selection of dietetic research articles to assess practice in the area.

## Methods

The purpose of this review is to evaluate the validity of claims made by weight loss researchers. A purposive sample was chosen: all articles published in *The Journal of Human Nutrition and Dietetics *between January 2004 and December 2008 in which the main focus was weight loss are included in the analysis. *The Journal of Human Nutrition and Dietetics *is the official journal of British Dietetic Association and as such is internationally regarded in the field of nutrition and dietetics. All articles are peer reviewed by two or more reviewers at least one of whom is a dietitian with a track record in publishing. Articles appearing in the journal can therefore be said to be representative of the best contemporary scientific research into weight management. The journal was hand searched and a total of 15 articles obtained. For ease of reference, these are indicated by the prefix 'J' where they are referred to throughout this article, eg. J[1]. The sample included an anniversary issue of the journal in which past influential papers were republished.

A narrative review was undertaken in which references to benefits or effectiveness of weight loss behaviour, or other justifications for intervention, in the selected articles were scrutinised for fitness for purpose. Effectiveness and other justification for intervention was chosen because a credible rationale for treatment is a prerequisite of ethical practice. Secondly, articles were reviewed for the rigour of reporting of adverse events and discussion of risk benefit. This is because it is an ethical requirement for recommendations for treatment to take account of any adverse effect and risk benefit. Thirdly, the appropriateness of any links made between recommendations and evidence were assessed. This was to ascertain to what extent recommendations accurately reflected research findings and to identify any role played by the dominant obesity discourse in influencing researchers' conclusions.

## Results

### Justification for treatment: implicit beliefs about weight loss and health

In the UK, a plethora of national anti-obesity documents recommend individual and population-level strategies to help people maintain a weight deemed healthy, usually categorised as having a body mass index (BMI) of 20-25. Weight management advice is typically justified by claims that weight loss will yield a range of benefits from improved metabolic fitness through to relief from musculoskeletal conditions and less discrimination. The belief in the value of weight loss is so firmly held that the rationale for intervention may not always be explicitly articulated. There is also concern that if no action is taken the population will become inexorably fatter.

This section investigates some of the common implicit justifications relied on when promoting weight loss. In several instances, a secular rise in population BMI is the sole justification for intervention J[3], J[4]. In other cases, the supporting rationale is high numbers of referrals for dietary weight loss and acknowledgment of treatment ineffectiveness J[5], J[6]. In the absence of further explanation or information on BMI and health, and likely outcomes of weight loss, these justifications rely on readers unequivocally accepting the dominant position on body weight, health and dieting, namely, that high BMI is a reliable indicator of poor health and weight loss a reliable remedy. This contrasts with the usual requirements of the scientific convention that assumes clear justifications for treatment are required.

### Justification for treatment: overall benefit

Obesity discourse, as reflected in the papers reviewed previously, provides examples of implicit justifications for treatment which are presented without an explicit, scientific rationale. In addition, it is not unusual to find claims of non-specific 'health benefits' which are not substantiated. In particular, the advice that clinically significant health improvement can result from a 5-10% loss of body weight is well rehearsed by researchers in the field and maintains a pervasive presence in anti-obesity campaigns and policy [7,8] and it is therefore not surprising to see it reiterated by studies included in this review.

The 5-10% weight loss target appears in a paper included in the review J[9] that reports a preliminary evaluation of men-only dieting groups. Authors claim that 'there are clear benefits to treating obesity' continuing that even modest weight reduction has beneficial effect. This latter claim is corroborated by citing Goldstein, 1992 [10], which as discussed in the following section, does not provide substantive justification for instituting weight loss interventions. The '5-10% weight loss' theme (in J9) is referenced to a document by the United Kingdom (UK) National Obesity Forum that claims health benefits but does not in turn supply any original source. Here, as illustrated elsewhere in the review, it appears that beliefs about weight and health acquire a truth status so that they circulate as intuitively appealing 'facts', immune from scrutiny and become used, and accepted by editors, without supporting references.

### Justification for treatment: regulation of blood lipids and blood pressure

More specific health benefits of losing 5-10% of body weight are given in anti-obesity documents and are repeated in some of the papers reviewed. Thus, a team of researchers from a UK anti-obesity research programme known as the Counterweight project J[11] recommend 5-10% weight loss for improvements in blood lipids, blood pressure, diabetes prevention and overall reduction in mortality.

In this section I will appraise three articles used by the Counterweight team to support claims that modest weight reduction improves blood lipid profiles and helps manage blood pressure. The authors of paper one [12] state that their meta-analysis indicates that weight reduction through dieting can normalise blood lipids. However, this conclusion contradicts their own observation that "it was impossible to identify the independent influence of dietary fat on changes in lipids and lipoproteins" (p 325). Moreover, further qualification is warranted due to the scope of the 70 studies included in their meta-analysis of which 67% had 20 participants or less, 35% lasted for 10 weeks or less and 82% had no control group.

A second paper [10] discussing the beneficial effects of weight loss on, among other indictors, blood pressure and blood lipids, is also inaccurately appropriated by the Counterweight project team. Studies on the effect of weight loss on blood pressure and blood lipids include those involving medication, exercise, salt restriction and other dietary modification. This means that the results do not in fact demonstrate the independent effects of weight loss on measured outcomes. As participant numbers in individual studies were typically small and studies were of short duration, a more circumspect interpretation of the results would seem justified.

The scientific merit of a third paper relied upon [13] can be discounted as authors neglected to include any methodology.

### Justification for treatment: Prevention of diabetes

Another common justification for weight management is its role in the prevention of diabetes [7], a claim I explore in this section.

Where the Counterweight project team J[11] makes claims for the role of weight loss *per se *in preventing diabetes, the cited articles are found wanting. The authors of one paper[14] note that their study does not demonstrate the effect of weight loss, as represented by the Counterweight project team, but demonstrates the effect of lifestyle intervention which included a significant increase in participants' exercise levels, a variable strongly associated with improvements in insulin resistance. Likewise, the second paper used by the Counterweight team [15] reports that ' The reduction in the incidence of diabetes was directly associated with changes in lifestyle' again including significantly increased exercise levels. A more detailed reading of the results indicates that, on the one hand, weight loss in the absence of change in exercise levels (change in other variables cannot be determined from the data presented so results cannot be confidently attributed to weight loss alone) did occur, with impact on incidence of diabetes. ('The odds ratio for diabetes in subjects in the intervention group who had lost more than 5 percent of their initial weight by the one-year follow-up visit was 0.3 (95 percent confidence interval, 0.1 to 0.7)'), However, changes in exercise levels led to more favourable outcomes:

Among the subjects in the intervention group who did not reach the goal of losing 5 percent of their initial weight, the odds ratio for diabetes in those who had achieved the goal with respect to exercise (more than four hours per week) during the first year was 0.2 (95 percent confidence interval, 0.1 to 0.6).

This finding is not further discussed by the Counterweight project team and so the benefits of health behaviour change are submerged in favour of weight-centred outcomes, a theme which I will develop later. In addition, there is no comment on the high quality evidence that weight loss at one year is almost invariably followed by weight regain [16].

### Effectiveness: success and failure in weight loss and health improvement

As I have discussed earlier, the dominant view in current obesity discourse asserts that weight loss *per se *is synonymous with improved health. This position is necessarily underpinned by the belief that weight loss is possible. The linked ideas, that weight loss is a reliable, effective method for improving health are the topic explored in this section.

Some studies included in the review used weight loss as an end in itself J[9] with no attempt to measure other specific clinical outcomes, change in diet and exercise behaviours, or to collect qualitative data on participants' views and experiences (or adverse effect). In this way, the discourse valorises the pursuit of thinness as a health goal in itself, paying little attention to the processes by which this may be achieved. This is problematic for three reasons.

First, the focus on weight loss as a primary goal overlooks the clinically significant benefits that can be expected through change to diet and exercise patterns independent of weight loss [17,18], for example, how a focus on weight obscures health benefits of improved fitness on diabetes prevention. Second, it detracts attention from the reality that weight loss may be achieved at the expense of health: Green and Buckroyd J[19] explain the role of disordered eating behaviours among 'successful' dieters. Third, the focus on attaining one specific body size over another raises ethical issues [20]. Researchers included in this analysis work within a particular framing of fatness as always pathological ie. 'obesity' and some articulation of the value judgements inherent in this position would help the reader assess scientific merit.

Some studies in the review that rely on weight change as the primary variable do not include details of any co-morbidity or explicitly refer to weight loss attempts by people who have no co-morbidities J[9],J[21]. In such cases the authors appear incognisant of the literature relating intentional weight loss in healthy people with increased mortality [22] as there is no discussion of the ethical tensions raised by the complexity of positions on weight management.

What about claims for effectiveness? The sample of papers included in this review had all the hallmarks of contemporary mainstream approaches to obesity in their approach to effectiveness. Hand in hand with a belief in the indisputable benefits of weight loss is the associated belief that clinically meaningful weight loss is a realistic goal for most people. This ignores the evidence of the overwhelming failure of weight reduction interventions: two high quality reviews find evidence that weight loss behaviour does not lead to long-term weight reduction and moreover that it can be counterproductive and harmful [16,23].

Despite this, the goal of sustained weight-loss was ubiquitously promoted as reasonable and desirable in all papers included in the review even though most papers also confirmed the high failure rate of weight loss in the long-term, a finding summed up as " there is increased awareness that short-term interventions are rarely successful' (p 504) J[24] and "long-term success in weight loss treatments for obesity is elusive" (p31) J[19]. Nevertheless, the collective supposition was that failure was due to lack of research/poor evidence base and there was no suggestion that it signalled the need to ultimately dispense with the belief in the success of weight loss.

A qualitative study of patients' views of dietary treatment for weight loss is a case in point. The study reports participants' views that, as compared to other people, they followed a healthy diet and were therefore at a loss to explain their current heavy weight and felt 'a lack of trust in their ability to succeed' (p491). One interpretation of this is suggested which speculates on a tension between patients assuming 'personal responsibility' for their health and the tendency for the patient to avoid responsibility through blaming their 'unfair' situation' (p 493) J[3]. Inherent in this analysis is the assumption that high BMI necessarily arises from 'excess' energy intake and can be 'corrected' by weight loss through energy deficit. This assumption ignores evidence that adult body weight is not primarily determined by current diet and exercise behaviours and is in fact highly resistant to alteration [25]. The framing also conflates weight outcomes with health and dietary quality. The erroneous belief that anyone can lose weight and keep it off if they try hard enough is later reiterated by the authors in a statement that dietitians 'still have far to go in enabling patients to achieve permanent weight loss' (p 493). The logic of this argument is faulty: the point is that permanent weight loss has been shown time and again to be an unattainable objective for the vast majority of dieters. It would seem, rather, that researchers still have far to go in recognising the significance of the evidence and responding accordingly.

Similarly, reporting on dietitians' views on weight management, Barr *et al. *J[24] wrote that the majority of respondents believed that 'loss of even a small amount of weight can be beneficial' ( p 509). In light of the rigorous debate about the evidence supporting this statement and the associated implicit belief that sustained weight loss is possible, and the statistically significant fact of weight regain, further discussion is merited.

### Effectiveness: use of energy deficit

It becomes apparent that mainstream obesity discourse is underpinned by the rationale that modifying energy balance will lead to weight loss, a view that is mirrored in papers in the review J[5], J[11] and discussed in this section.

Researchers variously calculated energy deficit from formulae derived from basal metabolic rate or from a diet history assessment. In one paper, where inconsistencies arose in actual *vs *expected weight loss, this was assumed to indicate 'non-compliance' as it was held that a 'linear relationship [exists] between weight loss and energy restriction' ( p 156) J[5]. As authors in the wider scientific community have noted 'Despite its scientific pedigree, the 'body as machine' model remains largely a theoretical proposition only. In fact, it is hard to find human beings in their 'natural settings' whose bodies conform to it.' (p.41) [26]. By continuing to advocate an energy deficit intervention without comment that it has never been proven to be effective long-term, and by automatically ascribing unexpected results to patient behaviour rather than exploring the model for potential flaws [25], there is a danger that the approach may be interpreted as being rather more successful than the evidence shows. This could compromise patient welfare and divert research capacity from more productive avenues.

A second paper by the Counterweight team also incorporated a tailored energy deficit diet J[11]. Authors state that, as compared to generalised low calorie diets, an energy deficit approach is a more effective method of assisting compliance and weight loss. The first paper cited to substantiate this claim is the paper mentioned above J[5]. This study followed patients for 3 months and involved small numbers. Regardless of any findings in relation to energy deficit diets/compliance/effectiveness therefore, it could be considered inappropriate to cite this paper as good evidential support for the intervention without qualifying its limitations. After all, as I have shown, many studies demonstrate that there is nothing unusual in participants losing weight over three months, the real test lies in maintaining weight loss - diet trials to date have consistently proven what is clinically ineffective. In fact, Frost *et al. *J[5] found there was no significant difference in actual mean weight loss with the energy deficit method except when a subgroup (n = 11) calculation was made.

The second paper cited by the Counterweight project team concludes that ' the [energy deficit] approach was no more effective in terms of weight loss than the 6279 kJ (1500 kcal) [generalised low calorie] approach (p1469) [27] and while compliance was indeed deemed better, weight regain was still significant. An odd, not say misleading, choice, to argue for the proven benefits of an energy deficit programme.

The Counterweight project team justify their treatment decision by underscoring the fact that the 600 kcal deficit diet is endorsed in a report from an expert obesity organisation, the Scottish Intercollegiate Guidelines (SIGN) [28]. Coming full circle, SIGN [29] cites Frost *et al. *J[5] to support their claims for the effectiveness of energy deficit. SIGN is a highly regarded document but the decision to extrapolate transferable claims for effectiveness from the short-term results of a subgroup of eleven people in a quasi-experimental trial and subsequently to use this as the basis for national recommendations is of dubious scientific merit. SIGN [28] further states that the energy deficit method *'*produces an average weight loss of > 5 kg in patients with a BMI of 30-43' (p24). Given the substantial amount of evidence available at the time that showed the overall ineffectiveness of non-surgical weight loss, this seems an extraordinary claim to make and it is notable that no reference is supplied. In the same vein, the suggestion that the status of evidence underpinning the energy deficit approach *'*'will probably be upgraded once the results of multiple international trials are published' (p 24) also appears rather fanciful. In short, the review illustrated a number of instances in the primary and secondary literature where citations justifying treatment and verifying effectiveness are unfit for purpose.

### Effectiveness: scientific credibility of claims for dieting success

Another common discursive device emerged in which authors made inflated proof claims that dieting in general was an effective strategy. A selection of examples follows. Herriot *et al. *J[21] state that a study [30] 'demonstrated the benefit of the inclusion of commercial weight programmes within the matrix of effective weight control behaviour' (p 79). While there is arguably room for ambivalent interpretation of the precise intended meaning of this phrase, it clearly points to a favourable outcome. The claim refers to the finding that a combination of decreased food quantity, cutting down on fats/sugars, use of a commercial weight loss programme and exercise led to a weight loss over two years of 0.03 kg compared to a group with no intervention (who gained weight) [30]. It is inconsistent with the data to subsequently propose that on this basis it 'seems likely that reputable commercial programmes could play an important role in any future national obesity strategy', notwithstanding the tentative qualifier 'could'.

Likewise, to say that 'all diets in 'Diet Trials' were ultimately successful in achieving weight loss in those who complied' ( p 78) J[21] is semantically true but does not accurately communicate the facts that 64% of people had withdrawn by week 8; that some people gained weight; that weight rebound was common. Further, it elides the authors' own informative discussion of the clinical (in)significance of weight loss in the short term. It would have been useful if the implications of a finding of short term weight loss were developed in relation to study outcomes, rather than, as was in fact the case, the earlier discussion being effectively overlooked in favour of a recommendation for more studies.

This overly optimistic tone, where a more qualified report of findings would increase scientific credibility, occurs in other papers. For example, one paper J[29] conveys an impression of reliable, positive outcomes in terms of long-term weight loss in concluding that *'*adding orlistat, sibutramine, exercise or behaviour modification to dietary advice can improve long-term weight loss' (p 293). But a more judicious reading of the results does not support this conclusion. First, in relation to sustained weight loss and medication the authors note that only one randomised controlled trial reported weight change post-medication and this found that weight regain was almost double that of the control (placebo) group. Second, in relation to behaviour therapy, the authors report that adding behaviour therapy to dieting led to weight loss at 12 months but weight increase at 60 months, indicating the need for clarification of the use of the phrase 'long-term'. Third, implicit in the claims for effective weight loss is the assumption of attendant health gains. However, authors note that 'few risk factors were examined in the non-drug trials, and trials which examined risk factors were usually small and therefore had insufficient power to detect clinically important differences' (p 312). Such differences could of course be positive or negative: orlistat, for example, was linked with an unfavourable change in blood HDL.

Some authors make explicit claims for the impact of dieting on clinical outcomes, other than weight change, which are similarly amplified. A systematic review of randomized controlled trials to determine the long-term benefits of weight reducing diets in adults by Avenell *et al. *J[31] that was included in the sample, is frequently cited in anti-obesity research. The title conveys the tacit assumption that diets do have long-term benefits. Authors note that adverse effects are frequently not reported but do not further discuss any practice implications or ethical dimensions of this omission which thus detracts from the applicability of their findings. The authors conclude that what they categorise as low fat diets are effective in achieving significant weight loss at 12, 24 and 36 months. It is further concluded that these diets have benefits in managing blood pressure, fasting glucose and lipids at 12 months and noted that there is limited data on risk factors after 12 months with 'a trend for blood pressure to be decreased by these diets at 36 months' (p 330). This statement appears to contradict an earlier statement in the results section that the limited data [from low fat diets] after 12 months no longer showed statistically significant risk factor changes [diastolic and systolic blood pressure, lipids and fasting glucose].

In the authors' discussion, the most recent paper cited in support of the claim that low fat diets can 'help prevent the development of diabetes, improve blood pressure control and reduce the use of anti-hypertensive medication for up to 3 years' is by Swinburn *et al. *[32] and this was therefore selected for appraisal. Swinburn *et al. *[32] report initial weight loss of a low magnitude (3.3 kg at 12 months) and note that 'like most interventions for weight loss, weight was regained in the long term' (p 622). Readers may consider this a salient fact, especially as data on long-term follow up is so sparse, but it is not reported in the paper under review. Swinburn *et al. *[32] note that compliant dieters showed reduced fasting glucose levels at 5 years. They explain that the low fat diet had significant effects on glucose tolerance at one year and that 'a smaller proportion of participants had type 2 diabetes or IGT [impaired glucose tolerance] in the [low fat] group (47 compared with 67%) [but] No intervention effect was present at 2, 3, or 5 years' (p 621). In this case, the paper shows a sub-group effect on fasting glucose levels but does not show diabetes prevention for up to 3 years, a claim it is used to corroborate.

Avenell *et al. *J[31] report the 20% difference in development of diabetes or impaired glucose tolerance found in Swinburn *et al. *[32] but do not provide details of when this finding was measured (ie. at 12 months) or subsequent lack of intervention effect which makes it impossible for readers to assess the relevance of the data. Swinburn *et al. *[32] discuss changes in exercise habits among study participants which is a variable relevant to fasting glucose levels and as such deserves mention, even if only to explain why it may not, in fact, have a bearing, in drawing conclusions of diet outcomes.

In a second paper included in the sample reviewed Dyson J[33] states that 'the health benefits of weight loss for overweight and obese people with type 2 diabetes are now well established', relying on a paper by Aucott *et al. *[34]. However, the paper by Aucott *et al. *does not substantiate the certainty of this claim - the authors caution that their conclusions are based on studies of moderate quality and find only two studies relevant to change in mortality following weight loss in overweight/obese people with diabetes. The results from one of the studies is non-significant. The other study was an observational study employing retrospective calculation of self-reported weight change which has inherent methodological limitations. The studies reporting changes in diabetic status after weight loss refer only to very fat, not 'overweight', people who underwent weight loss surgery. In one study the follow-up period of the post-operative changes in glucose handling was not specified and no data was provided from those patients who were available for follow-up. There is a similar lack of useful data in the second paper. In short, the paper by Aucott *et al. *[34] reports that one large observational study found reduced mortality with intentional weight loss in overweight/obese people with type 2 diabetes and poorly specified post-operative improvements in glucose handling in ' morbidly obese' people with diabetes. It is doubtful whether this qualifies as firmly establishing the health gains of weight loss in diabetes.

Overall, throughout the reviewed papers, authors typically comment on the lack of information on changes in risk factors and quality of life from the currently available evidence of weight loss trials. In addition, one team discusses the underreporting of obesity and low treatment rates in primary care. These authors speculate that 'lack of evidence of the effectiveness of weight management interventions in primary care' may be a contributing factor J[35]. There is, however, ample evidence that points to treatment ineffectiveness [16]. It is a logically flawed argument to presuppose that it is only a matter of time until effectiveness is demonstrated.

### Adverse Effect

In the third section of this paper I will consider reporting on adverse effects in more depth. Generally, authors included in the review represent the effects of weight fluctuation on health more neutrally than in the wider literature. Thus, it is suggested J[24] that although there is evidence that weight fluctuation may increase risk factors for heart disease, 'it is premature to make strong conclusions about effects on physical health'. A reference is given which investigates links between weight cycling, blood lipids and blood pressure. This position contrasts sharply with the British Nutrition Foundation's statement [36]:

"a positive association has consistently been observed between body weight fluctuation and all-cause mortality and usually... with coronary mortality in particular. This finding is very robust, further confirmation is found in the British Regional Heart Study (Wannamethee & Shaper, 1990), in the Seven Nations Study (Peters *et al.*, 1995) and in the Iowa Women's Health Study (French *et al.*, 1997) (p 137)."

Some discussion of the controversy would have enhanced the credibility of the authors' argument. Further, the impact of weight fluctuation on physical health is considered in relation to cardiovascular parameters only. Omitting discussion of the detrimental effects of weight fluctuation on bone health [37] weakens the scientific legitimacy of the argument. A more detailed and balanced discussion of the general literature on weight fluctuation and health may be necessary to improve the validity of their argument.

Authors of a second paper in the sample report findings that study participants intended to diet in future on an *ad hoc *basis J[21] which raises the issue of weight fluctuation. There is no discussion of how the ethical issues this presented to the researchers were dealt with and authors do not flag up the consequences of weight cycling to participant health.

Similarly, in a third paper J[19], the controversy over weight fluctuation is elided in a statement that 'the health impact of weight loss has been found to be more strongly associated with net amount of weight loss as opposed to the pattern of weight change over time' (p 33 ). The article displays a positive attitude towards weight loss and it may be reasoned that the reader is expected to share the assumption that the 'health impact' referred to is a beneficial impact rather than a detrimental one. There is no discussion of the adverse effects of dieting on physical health which limits confidence in the authors' familiarity with the literature in their area and consequently in the clinical validity of their recommendations. It would be helpful if the authors highlighted that this was an area of considerable debate in the same way that they draw attention to conflicting views and findings concerning weight management and eating cognitions and behaviours. The same authors state both that 'long-term success in weight management treatments for obesity is elusive' (p 31) and also imply that dieting typically leads to overall weight loss by describing a pattern of weight loss followed by regain of some, but not all, of the initial weight loss. This contradiction warrants further expansion as it is in stark contrast to high quality findings on the long-term outcomes of dieting [16] which have greater validity than the study [38] later relied on to support claims for weight loss maintenance. Clearer demarcation between opinion and evidence to increase transparency in the development of recommendations may improve the transferability of results to practice and improve patient welfare.

There are other instances, aside from consideration of adverse effect, where authors omit relevant debate. Jehn *et al. *J[4], for example, refer to the findings of the United States National Weight Control Registry on variables associated with long-term weight loss. It would have been useful if they had alerted the reader to some of the challenges raised to these claims [39].

## Discussion

### Health at Every Size

I have argued that weight management cannot be considered to be an evidence based intervention. Moreover, a focus on individual behaviour change in the pursuit of health detracts attention from the wider social and material determinants of morbidity and mortality. Further, the weight-centred paradigm stymies research and practice developments into a more effective, alternative approach to improving nutritional wellbeing. This non weight-centred approach is known as 'health at every size', or HAES. (HAES is also known as a no-diet or size acceptance approach).

HAES advocates adopt a weight-neutral approach to lifestyle change where the primary goal is moved from achieving weight change to modifying health behaviours. Outcome measures relate to metabolic fitness - such as blood pressure and cholesterol - eating disorder symptomology, exercise levels, and psychological parameters. HAES research demonstrates that clinically and statistically significant improvements in a range of health measures can be achieved independently of weight reduction [17,18]. That is, improving someone's health behaviours has health benefits even when weight stays the same and moving the focus off weight reduction helps people sustain improved health behaviours. For example, in a small randomised controlled trial [18] (n = 78) comparing HAES with conventional weight management, HAES is associated with sustained improvements in health outcomes at 2 years follow-up, with weight stability, and with no adverse effect, whereas early improvements were not maintained in the diet arm of the trial, and there was a decrease in self-esteem. This paper is a classic in HAES scholarship and the research results are typical of findings in other HAES research [17].

There are four key tenets of HAES that mark it as different from conventional healthy weight interventions. First, it is weight neutral; second, it teaches people to rely on internal signals and to eat intuitively, rather than relying on external regulation to eat; third, it encourages positive body esteem in people of all shapes and sizes (size acceptance). Importantly, HAES challenges implicit and overt size discrimination in and beyond the clinic.

### Implications for research and practice

It has been suggested that 'a temptation in clinical research is to sacrifice the interests, [and] the health' of research participants (p 69) [40]. This review indicates that anti-obesity researchers publishing in contemporary UK dietetics adopt a particular stance towards body weight management practices that may inadvertently compromise the health of participants. Weight management research appears to occupy a hallowed place where deviations from regular scientific conduct are readily tolerated, for example, continued support of research programmes that do not adequately report adverse effect, or rely on acceptance of common assumptions that are inadequately supported by data, which in turn may point to a lack of stringency in research ethics decisions regarding weight management. It also demonstrates the influence of ideology in selecting and reporting data, whether intentional or not, which has material implications for research participant/patient welfare and research directions in that it eclipses other positions and ensuing discussions are poorer for this bias.

The papers analysed in this review unfailingly frame fatness as a pathological condition that is primarily under personal control through volitional modification of eating and exercise habits. A broader reading of the literature shows that there is in fact a vigorous scientific controversy about many of the treatment premises that are taken for granted within this dominant anti-obesity agenda. This wider debate interrogates the theory and science behind assumptions about the links between body mass index/body weight (specifically fatness) *per se *and health outcomes [1,17,26,41-44].It also questions the appropriateness of using anthropometric measurement in diagnosing disease/risk and the value of BMI as a meaningful variable for assessing research outcomes. These critical perspectives on claims made about an obesity crisis further explore the broader ethical consequences of the thinness imperative. As seen in this review, dietetic papers on weight management conventionally conclude with recommendations for more robust and informative trials. I would instead suggest that there is more progress to be made from reframing the scope of enquiry. Within this reframing further research is needed to determine: the appropriateness of criteria relied on to determine the scientific justification for weight reduction research; the completeness of Patient Participant Information Sheets submitted to research ethics committees; the impact of size bias on clinical decision making (see also [42]); the safety and effectiveness of treatments that seek to improve cardiovascular risk factors independent of weight change; reasons for intransigence in practitioner and researcher commitment to prescribing weight reduction; the effectiveness of interventions designed to improve health professional's practice with regard to evidence based intervention in nutritional lifestyle intervention; dietetic resistance to adopting the evidence-based health at every size paradigm [2]. This review also has ethical implications regarding researcher and peer reviewer responsibility and accountability in the use of citations to support claims.

## Competing interests

LA promotes a health at every size approach to nutritional intervention. She has no competing financial interests.

## Authors' contributions

LA researched and wrote the paper.
